# Major life events, stress appraisal, and migraine: results of the American Migraine Prevalence and Prevention (AMPP) study

**DOI:** 10.1186/1129-2377-14-S1-P9

**Published:** 2013-02-21

**Authors:** AN Manack, D Serrano, CC Turkel, RB Lipton, DC Buse

**Affiliations:** 1 Allergan, Inc., Irvine, California, USA; 2 Vedanta Research, Chapel Hill, North Carolina, USA; 3 Allergan, Inc, USA; 4 Department of Neurology, Albert Einstein College of Medicine and the Montefiore Headache Center, Bronx, New York, USA

## Objective

To assess cross-sectional differences among persons with chronic migraine (CM) versus episodic migraine (EM) in major-life-events (MLE) rates and appraisal of events as stressful (SLE).

## Methods

AMPP is a longitudinal, US-population-based study for which questionnaires were mailed to 24,000 severe headache sufferers and followed annually. Respondents with ICHD-2 migraine were stratified as either CM (¡Ý15 headache-days/month) or EM (<15 headache-days/month). MLE occurrences were defined as moving, change in significant-relationship status, work/school stressors, events w/children, deaths, other over preceding year. For endorsed MLEs, respondents were asked to assess stress level on a 5-point Likert scale (1=¡°not at all¡±/5=¡°very¡±). To identify a SLE, responses were dichotomized with a cut-score of ¡Ý4. Ordered logistic regressions used to model odds of reporting more SLEs. Results In 2007, 14,069 individuals responded and 557 had CM and 748 had EM. 80.1% of CM reported ¡Ý1 MLE in preceding year vs 78.6% of EM. The proportion of CM vs EM reporting no MLEs (18.2 vs 21.4%), 1 MLE (26.3 vs 27.5%), 2 MLEs (27.8 vs 25.1%) or ¡Ý3 MLEs (27.8 vs 25.1%) revealed more MLEs for CM. 76.5% of CM reported ¡Ý1 SLE in preceding year vs 71.4% of EM. Proportion of CM vs EM reporting no SLEs (19.3 vs 23.3%), 1 SLE (32.9 vs 34.1%), 2 SLEs (27.2 vs 25.2%) or ¡Ý3 SLEs (20.7 vs 17.4%) revealed more SLEs for CM. Unadjusted odds ratio (OR) comparing those with stress scores 4&5 w/lower scores was ~25% greater for CM vs EM (OR=1.25, 95%CI 1.04-1.49). Adjusting for age, gender and race produced similar

## Results

(OR=1.27, 95%CI 1.07-1.52).

## Discussion

CM persons reported more MLEs in the preceding year and were more likely to perceive events as stressful. Longitudinal analyses are required to assess whether MLEs/SLEs are risk factors for¨Cor consequences of¨CCM.

## Funding

The AMPP study was funded through a research grant to the NHF from Ortho-McNeil Neurologics. Additional analyses were supported by Allergan, Inc.

